# The mechanism of PDE7B inhibiting the development of hepatocellular carcinoma through oxidative stress

**DOI:** 10.3389/fimmu.2024.1469740

**Published:** 2024-11-21

**Authors:** Yunfeng Luo, Huaide Gao, Jianghua Zhao, Lin Chen, Jianguo Shao, Linling Ju

**Affiliations:** ^1^ Affiliated Nantong Hospital 3 of Nantong University, Nantong Third People’s Hospital, Nantong, Jiangsu, China; ^2^ Medical College of Nantong University, Nantong University, Nantong, Jiangsu, China; ^3^ School of Health Medicine, Nantong Institute of Technology, Nantong, Jiangsu, China

**Keywords:** hepatocellular carcinoma, PDE7B, proliferation, invasion, migration

## Abstract

**Background:**

Liver cancer presents a significant challenge to global health and is currently ranked as the sixth most common form of cancer worldwide. Recent research indicates that phosphodiesterases play a role in various physiological and pathological processes, with a specific focus on their impact on cancer advancement. There is a scarcity of studies investigating the function and mechanisms of phosphodiesterases in the development and progression of hepatocellular carcinoma (HCC).

**Methods:**

Real-time fluorescence quantitative polymerase chain reaction (qRT-PCR) and Western blotting were employed to analyze the expression of PDE7B in hepatocellular carcinoma tissues and cells. The biological role of PDE7B in HCC was investigated by both overexpressing and knocking down PDE7B in liver cancer cell lines. Furthermore, potential target proteins of PDE7B were identified through transcriptome sequencing.

**Results:**

PDE7B is conspicuously reduced in tissues and cells of hepatocellular carcinoma, showing a connection with an unfavorable prognosis. Inhibiting PDE7B boosts the growth, movement, and infiltration of liver cancer cells, while its increased expression has the reverse impact. According to our trials relating to oxidative stress, PDE7B appears to control cell death in liver cancer cells by impacting the production of reactive oxygen species. Therefore, we propose that PDE7B could hinder the initiation and advancement of HCC through an oxidative stress pathway.

**Conclusion:**

The research we conducted reveals that PDE7B, a gene with minimal levels of activity in hepatocellular carcinoma, possesses the capacity to inhibit the proliferation, invasion, and migration of HCC cells. PDE7B can impact the development of hepatocellular carcinoma by adjusting mechanisms related to oxidative stress.

## 1 Introduction

Primary liver cancer represents a substantial global health challenge and currently stands as the sixth most prevalent cancer type worldwide. Additionally, it is the third leading cause of cancer-related mortality (1, 2). Hepatocellular carcinoma (HCC) constitutes the majority of primary liver cancer cases, accounting for 85% to 90% of incidences (3–5). The incidence of HCC is strongly influenced by two predominant global risk factors: hepatitis C virus (HCV) and hepatitis B virus (HBV) (6). Early-stage HCC is often difficult to detect and has almost no clinical symptoms. Most patients diagnosed with HCC have almost all entered the middle or late stage. Currently, treatments for advanced HCC patients are limited (7). Due to the limited availability of standard chemotherapy options, the treatment of HCC patients has primarily depended on targeted therapies (8, 9). However, the emergence of immunotherapy has broadened the treatment landscape for advanced HCC (10). Despite advancements in the management of HCC, the overall survival rate for patients with advanced-stage liver cancer remains low. To address this issue, it is essential to further explore new specific biomarkers to enhance the early diagnosis of HCC and ultimately improve patient survival rates.

HCC has a complex genetic background characterized by somatic mutations, copy number variations, and epigenetic modifications (11). Cyclic nucleotide phosphodiesterases (PDEs) are a group of proteins pivotal in modulating intracellular levels of cyclic adenosine monophosphate (cAMP) and/or cyclic guanosine monophosphate (cGMP) via their phosphohydrolase function (12–14). cAMP and cGMP are intracellular second messengers in human cells and play critical roles in regulating cellular activities (15). PDEs can degrade intracellular cAMP and cGMP, consequently terminating the biochemical impacts facilitated by a second messenger (16). These impacts include cell proliferation, differentiation, apoptosis, cell cycle, and various other physiological processes (17). PDEs are categorized into 11 distinct families, encoding PDE1-PDE11, which include 21 different subtypes and splicing mutants, 16 of which can regulate cAMP levels (18, 19). The PDE7 subfamily contains two subtypes, namely PDE7A and PDE7B (20). PDE7B is extensively expressed in various organs including the pancreas, heart, brain, thyroid, skeletal muscle, and liver (21). One significant characteristic of PDE7B is its strong attraction towards cAMP, indicating its involvement in cAMP signaling pathways (22). Most eukaryotic cells separate the regulatory subunit (C) and catalytic subunit (R) of protein kinase A (PKA) with increasing intracellular cAMP concentration (23). Next, the phosphorylation of numerous targets by protein kinase A, a key regulator of effector enzymes and ionic channels, including but not limited to CREB, Raf, Bad, etc., can stimulate gene transcription (24). This process is crucial for cellular growth and differentiation. In recent years, PDEs, as new potential therapeutic targets, have attracted widespread attention from many investigators and have become a new research focus.

While the role of PDEs in cancer has been widely studied, the specific function of PDE7B in HCC has not been thoroughly explored. Our study represents one of the first comprehensive investigations into the expression and functional role of PDE7B in HCC. Our research focuses on revealing the potential regulatory role of PDE7B in HCC, particularly its unique involvement in hepatocyte proliferation, apoptosis, and signaling pathway regulation. By combining clinical sample analysis with both *in vivo* and *in vitro* experiments, we are the first to comprehensively evaluate the heterogeneity of PDE7B expression in HCC and its association with patient prognosis. This opens up new possibilities for the development of targeted therapies.

## 2 Materials and methods

### 2.1 Clinical data and specimens

Between 2018 and 2022, radical hepatectomy patients at the Department of Hepatobiliary Surgery of the Third Peoples Hospital of Nantong City provided HCC and adjacent nontumor tissues for analysis. After collecting the specimens, the specimens were cut and stored at -80 °C until use. All individuals included in this study provided informed consent. The Ethics Committee of Nantong Third People’s Hospital granted ethical approved for the research in accordance with the Declaration of Helsinki.

### 2.2 Gene expression analysis

The analysis of PDE7B mRNA expression was obtained from the TCGA and GSE databases. To validate the protein expression level of PDE7B, western blotting and immunohistochemical analysis were performed on HCC tissues and normal liver tissues.

### 2.3 Cell culture

The normal human liver LO2 cell line and HCC cell lines (Hep3B2.1-7, HuH-7, Li-7, and PLC/PRF/5) were provided by the Chinese Academy of Science (Shanghai, China). LO2, Hep3B2.1-7, and Li-7 cells were cultured in RPMI 1640 medium (Gibco, USA), while HuH-7 and PLC/PRF/5 cells were cultured in MEM (Gibco, USA) supplemented with 10% fetal bovine serum (Gibco, USA). All cells were maintained in a 37 °C incubator with 5% CO2. In HCC cell lines, PDE7B exhibits relatively high expression levels in the PLC/PRF/5 cell line, while its expression is comparatively low in the Hep3B2.1-7 cell line. Consequently, we selected the PLC/PRF/5 cell line for knockdown experiments and the Hep3B2.1-7 cell line for overexpression experiments.

### 2.4 Transfection

We designed and synthesized small interfering RNAs (siRNAs), overexpression plasmids, and lentiviruses targeting PDE7B by GenePharma (Shanghai, China). The sequence of siRNA was listed in Supplementary Material. Temporary modifications were introduced by transfecting siRNAs into PLC/PRF/5 cells and plasmids into Hep3B2.1-7 cells using Lipofectamine 3000 (Invitrogen, USA), following the manufacturer’s instructions. We determined the transfection efficiency by performing qRT−PCR analysis.

### 2.5 RNA preparation and qRT−PCR

Total RNA from tissues and cells was extracted using TRIzol reagent following the manufacturer’s instructions. Subsequently, cDNAs were synthesized from the extracted RNA using the Prime-ScriptTM RT reagent kit (Takara, Dalian, China). RNA concentration was assessed using a NanoDrop 2000c spectrophotometer (Thermo Fisher Scientific, Waltham, MA, USA) before cDNA synthesis. SYBR^®^ Green PCR Master Mix was utilized for cDNA amplification, and qRT-PCR was conducted on a CFX96 Deep Well Real-Time System (Bio-Rad, CA, USA). The expression levels of the target genes were calculated using the 2^−ΔΔCt method. The internal control chosen for normalization was glyceraldehyde-3-phosphate dehydrogenase (GAPDH).

### 2.6 Western blotting

After transfection for 72 hours, RIPA buffer (Beyotime) was used to extract proteins from the tissues and cells. Protein concentration was assessed using the BCA Protein Quantification Kit (Vazyme) with a NanoDrop 2000c spectrophotometer (Thermo Fisher Scientific). SDS−PAGE was used to separate equivalent quantities of protein extracts, which were subsequently transferred onto PVDF membranes. Following a 2-hour blocking step in 5% nonfat milk, the membranes were incubated overnight at 4 °C with the following primary antibodies: GAPDH, CREB, p-CREB, HRP-conjugated-β-actin, PDE7B, N-cadherin, E-cadherin, vimentin, PI3K, p-PI3K, AKT and p-AKT. The antibodies used were acquired from Proteintech (Wuhan, China). Subsequently, the membranes were subjected to 1-hour treatment with HRP-conjugated secondary antibodies at room temperature, followed by extensive washing. Finally, the membranes were exposed using an automated exposure machine (Clinx ChemiScope).

### 2.7 Migration and invasion assays

We transfected siRNA into PLC/PRF/5 cells and transfected the plasmid into Hep3B2.1-7 cells, subsequently placing both cells in a culture incubator for incubation. After 48 hours of transfection, 1 × 10^5^ cells were suspended in medium devoid of serum and subsequently seeded into 24-well plates coated with Matrigel for the invasion assay. In contrast, the migration assay required the use of plates without Matrigel. The tests were carried out according to the instructions. The bottom of the plate was supplemented with 600 µl of MEM medium supplemented with 20% FBS to attract the cells to the chambers. The cells were fixed using 4% paraformaldehyde and stained in crystal violet to indicate migrating or invading cells, respectively. The cells were subsequently observed under a light microscope, and the cell count was determined using ImageJ software.

### 2.8 5-Ethynyl-20-deoxyuridine cell proliferation assay

Cell proliferation was measured in accordance with the manufacturer’s instructions, utilizing an EDU detection kit provided by RiboBio. After a two-hour incubation period, the cells were fixed and subjected to staining. Finally, the cells were visualized by employing a fluorescence microscope. The image processing methods were applied to every pixel in the image and the changes didn’t alter the information illustrated in the figure.

### 2.9 Colony formation assay

After 48 hours of transfection, the PLC/PRF/5 and Hep3B2.1-7 cells were seeded into 6-well plates, each containing 3000 cells. Subsequently, the cells were fixed using paraformaldehyde, and after incubating for 14 days, they were subjected to crystal violet staining. Under a light microscope, the colonies were observed, visualized, and enumerated.

### 2.10 Cell apoptosis assay

The cells that underwent transfection were collected and incubated with Annexin V-PE/7-AAD solution for 15 minutes. The experimental procedure followed the instructions outlined in the Apoptosis Detection Kit protocol. Subsequently, the rate of apoptosis was measured using a flow cytometer, and the data obtained were analyzed using FlowJo VX software.

### 2.11 Mitochondrial superoxide assay

The cells were treated with MitoSOX Red reagent (Thermo Fisher Scientific). To prepare a working solution, a 5 mM stock solution of MitoSOX reagent was diluted in HBSS/Ca/Mg to achieve a concentration of 5 µM. Subsequently, 2 ml of the 5 µM MitoSOX reagent working solution was added to a 6-well plate, and the cells were incubated at 37 °C for 10 minutes while shielded from light. Flow cytometry was used to analyze the cells following the manufacturer’s instructions.

### 2.12 cAMP and PKA assays

A Cyclic AMP Assay Kit and PKA Assay Kit were used to assess cAMP and PKA levels, respectively. The cells transfected with the control, PDE7B siRNAs, or PDE7B plasmids were incubated in 96-well plates at 1 × 10^4^ cells per well. The assay was performed per well. The assay was conducted according to the manufacturer’s instructions.

### 2.13 Hematoxylin and eosin staining and immunohistochemistry staining

Liver tumors and adjacent normal liver tissues, cut to 4 mm thickness, were fixed in formalin and embedded in paraffin. To assess PDE7B expression, we conducted H&E and IHC staining with an anti-PDE7B monoclonal antibody following the manufacturer’s instructions. Microscopic imaging was employed to acquire the resulting images.

### 2.14 Animal experiments

All animal experiments were approved by the Ethics Committee of Nantong University. LV-NC and LV-PDE7B were transfected into Hep3B2.1-7 cells. A total of 1 × 10^7^ HCC cells mixed with Matrigel were injected into the skin of mice near the lower limb. Tumor size was assessed every 72 hours for 21 consecutive days. Subsequently, the mice were euthanized, and the tumors were extracted. Later, the tumors were preserved using paraformaldehyde and subjected to H&E and IHC analyses. All animal experiments used in this study were approved by the Medical Ethics Committee of Nantong University and in line with the guidelines for Animal Care of the National Institutes of Health.

### 2.15 RNA sequencing

To identify potential target proteins of PDE7B, we employed high-throughput sequencing and bioinformatics analysis. CapitalBio Technology (Beijing, China) was also involved in this task. Additionally, we scrutinized the target genes and performed GO and KEGG enrichment analyses.

### 2.16 Statistical analysis

At least three repetitions were conducted for each experiment. The software packages utilized for data analysis included GraphPad Prism and SPSS. We selected the Student’s t-test and Kaplan-Meier survival curve analysis based on their effectiveness and widespread use in relatively small sample datasets. The Student’s t-test is a classical method for detecting mean differences between two groups, which adequately meets the needs of this study. For survival analysis, we chose Kaplan-Meier curves and the log-rank test as they are widely accepted standard methods for comparing survival times, especially with smaller sample sizes in medical data. To assess disparities between two groups, Student’s t- test and one-way analysis of variance (ANOVA) were employed for comparisons among multiple groups. A p-value less than 0.05 denoted statistical significance.

## 3 Results

### 3.1 PDE7B expression was downregulated in HCC

**Figure 1:**
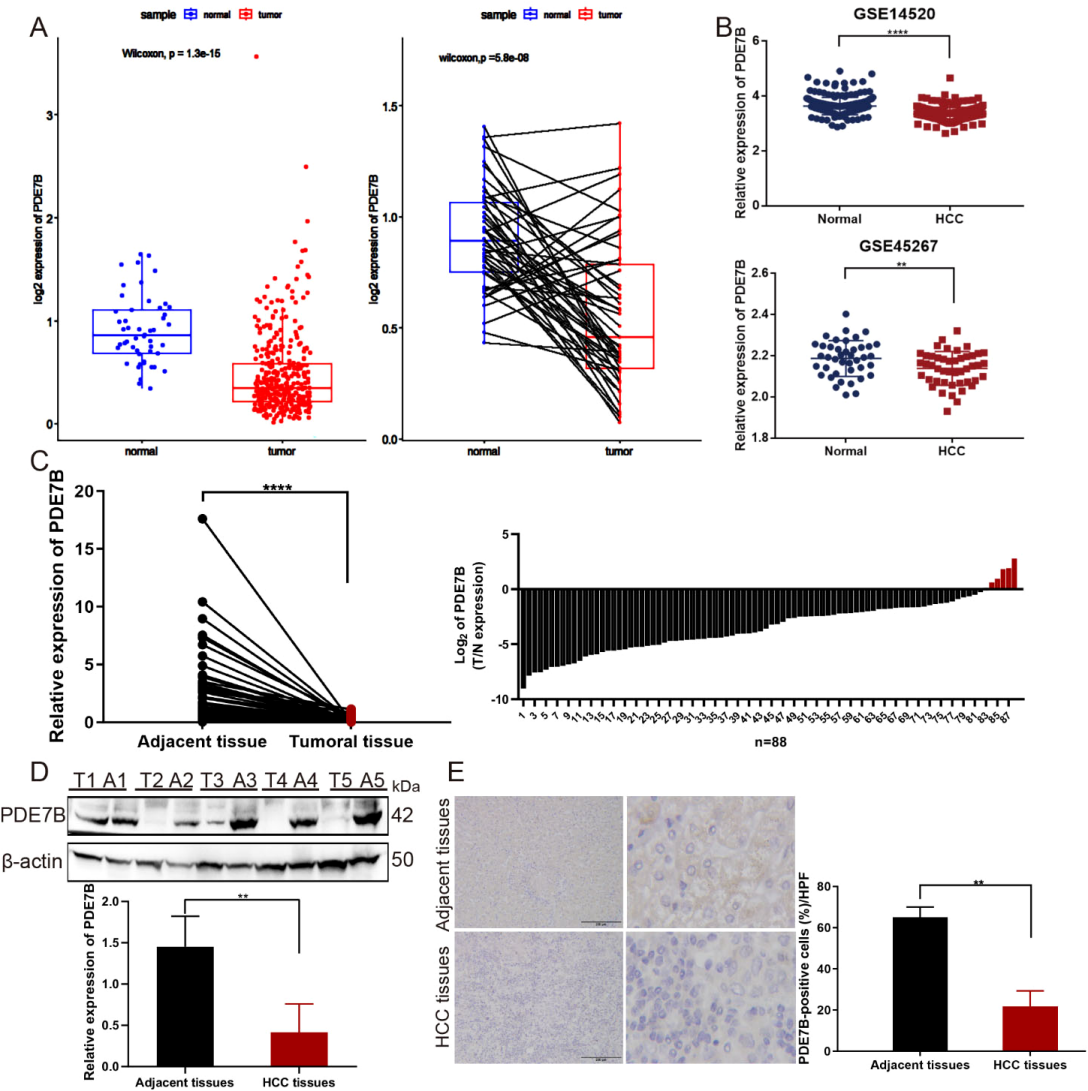
The expression of PDE7B in cancer. **(A)** PDE7B expression was markedly lower in HCC tissues compared to normal tissues according to the TCGA database. **(B)** The expression levels of PDE7B in GSE14520 and GSE45267 cohorts. **(C)** qRT-PCR analysis quantified PDE7B mRNA expression levels in hepatocellular carcinoma tissues (n=88) and adjacent tissues. The error bars in the figure represented the standard error of the mean. Each experiment was conducted in triplicate. The protein levels of PDE7B were detected in HCC tissues and normal controls by western blot **(D)** and IHC staining **(E)** (*p<0.05).

To explore the association between PDE7B expression and the status of patients with HCC, RNA sequencing data derived from both tumor and nontumor tissues obtained from the TCGA database and from the GSE14520 and GSE45267 cohorts were analyzed (**Figures 1A, B**). These results suggest a notable decrease in PDE7B expression levels in hepatocellular carcinoma tissue samples compared to normal tissue samples. Moreover, we confirmed that the mRNA expression level of PDE7B was downregulated in paired HCC tissues from our own cohort (**Figure 1C**). Finally, western blot analysis was confirmed that the PDE7B protein was expressed at lower levels in HCC tissues (**Figure 1D**). Consistent with these findings, IHC staining was employed to confirm the reduced expression of PDE7B protein (**Figure 1E**). Taken together, these results revealed that PDE7B is observably downregulated in HCC patients and may be related to the development of HCC.

### 3.2 PDE7B expression is correlated with clinical parameters and prognosis in HCC

**Figure 2:**
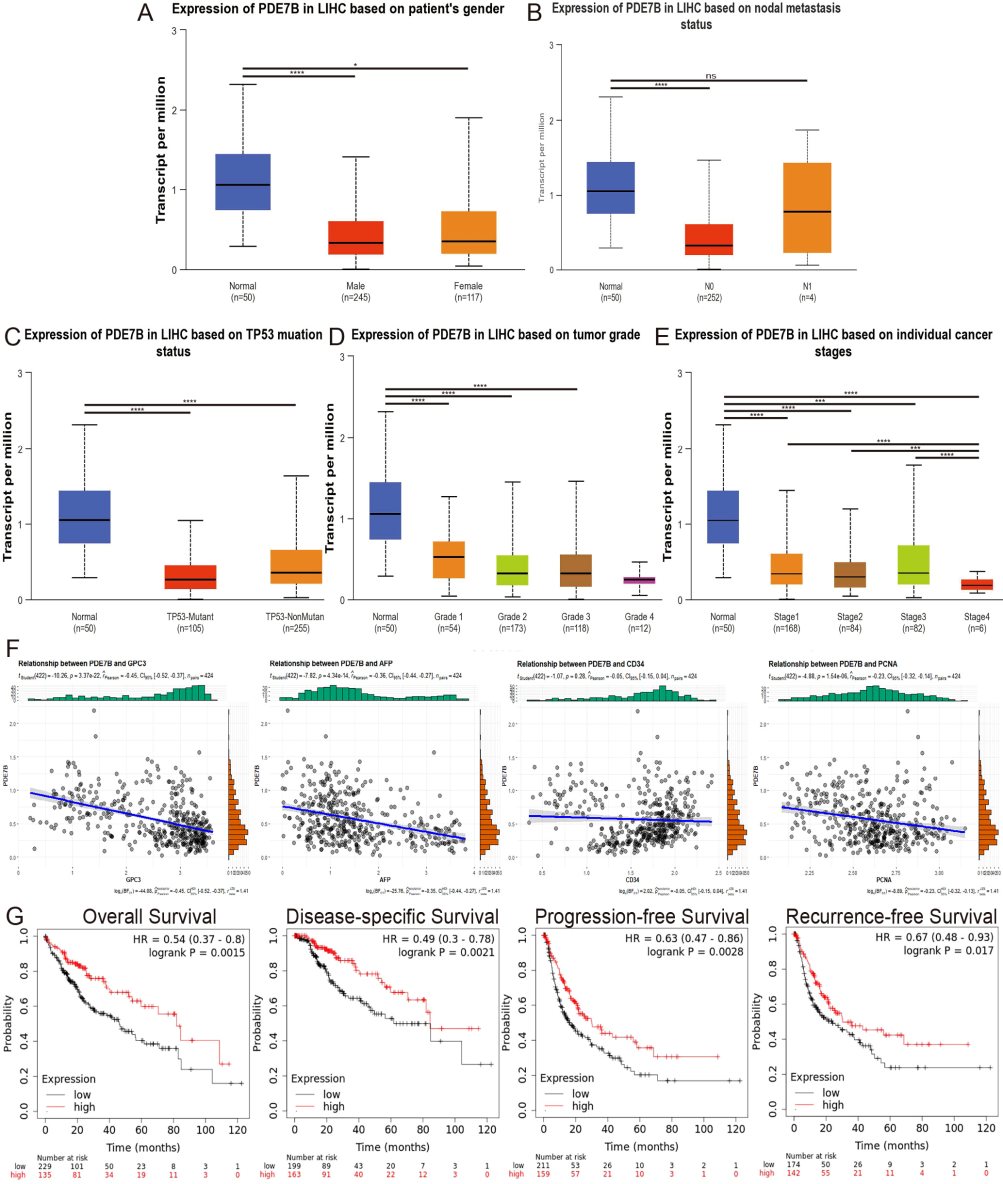
The correlation between different clinical parameters and PDE7B expression and the prognosis of patients with HCC stratified by PDE7B expression status. The UALCAN database showed that PDE7B is associated with patient sex **(A)**, nodal metastasis status **(B)**, TP53 mutation status **(C)**, tumor grade **(D)**, and individual cancer stage **(E)**. **(F)** Relationships between PDE7B expression and GPC3, AFP, CD34 and PCNA expression. **(G)** Correlations of PDE7B expression with OS, DSS, PFS and RFS in HCC patients according to the K-M plotter database (*p<0.05).

To determine the significance of PDE7B in hepatocarcinoma, we investigated the association between PDE7B expression and clinical characteristics utilizing the UALCAN database. Patients with HCC were categorized into various subsets based on sex, TP53 mutation status, nodal metastasis status, tumor grade, and individual cancer stage. Despite the absence of any noteworthy variation in the level of PDE7B expression according to patient sex, nodal metastasis status, or TP53 mutation status, there was a noticeable decrease in PDE7B expression in HCC tissues compared to corresponding normal tissues (**Figures 2A–C**). Compared to that in the control group, PDE7B expression in Grade 1, 2, and 3 tumors was significantly lower, depending on the tumor grade. Moreover, PDE7B expression in Grade 4 patients was notably lower than in the normal control group; however, this disparity did not reach statistical significance (**Figure 2D**). PDE7B expression was significantly downregulated in patients with individual cancer stages 1, 2, 3 and 4. In addition, compared with that in the Stage 1, 2, and 3 groups, the PDE7B expression level in the Stage 4 group was markedly lower (**Figure 2E**).

According to the online database, PDE7B expression is significantly negatively correlated with GPC3, AFP, and PCNA expression in patients with HCC. However, no significant correlation was found between PDE7B and CD34 (**Figure 2F**). We investigated the influence of PDE7B expression on the survival curves of HCC patients using the Kaplan-Meier Plotter database. Using the median expression level of PDE7B as a reference point, individuals diagnosed with HCC were categorized into groups according to high or low PDE7B expression. An investigation of survival rates demonstrated that individuals with lower PDE7B expression exhibited significantly improved outcomes in terms of disease-specific survival (DSS), overall survival (OS), recurrence-free survival (RFS), and progression-free survival (PFS) (**Figure 2G**). These findings clearly demonstrated a strong association between PDE7B expression, clinical indicators, and HCC prognosis.

### 3.3 PDE7B affects the migration, invasion and proliferation ability of HCC cells *in vitro*

**Figure 3:**
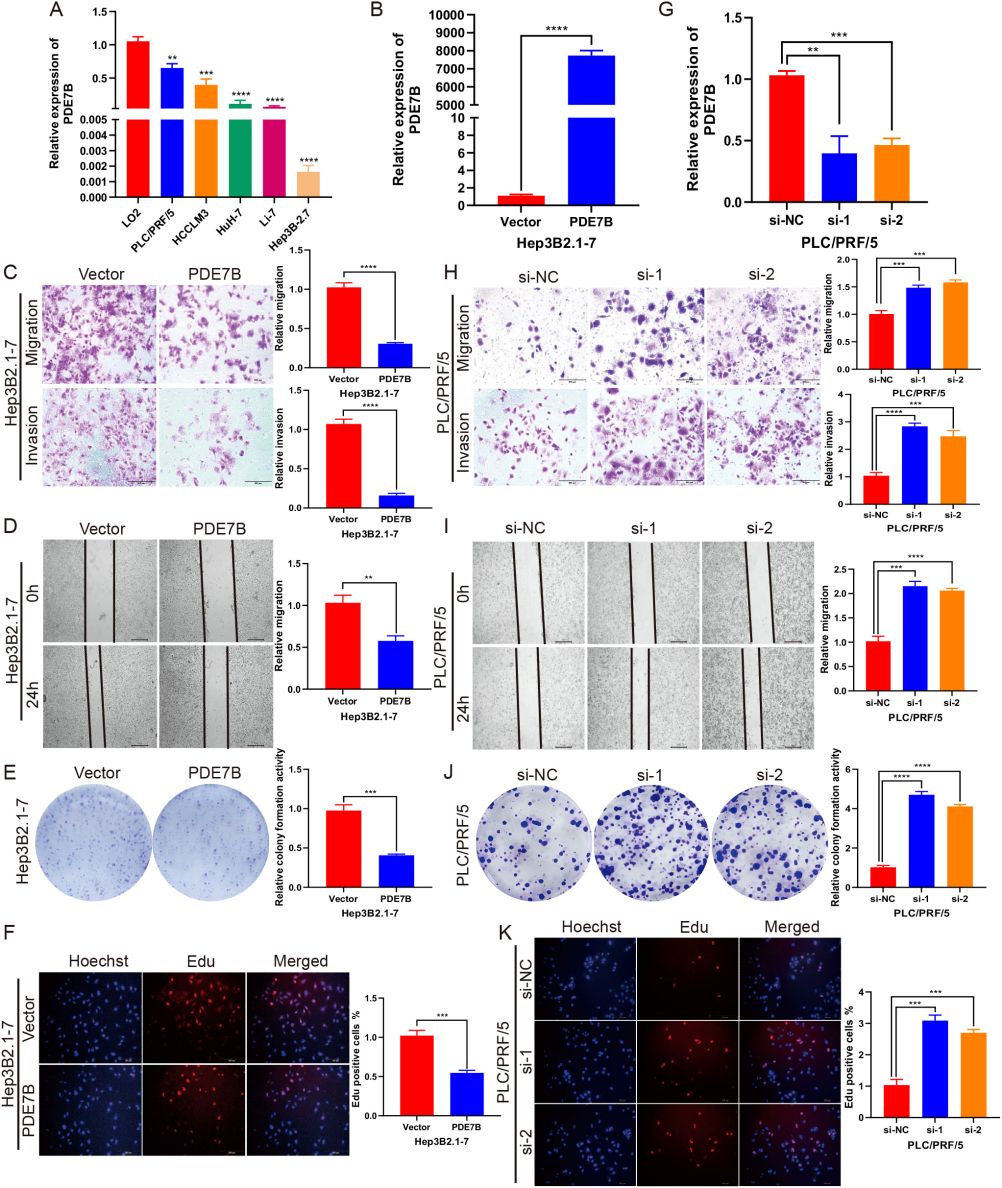
PDE7B affects cell migration, invasion and proliferation. **(A)** PDE7B expression was measured by qRT−PCR in HCC cell lines and LO2 cells as a control. **(B)** Transfection efficiency of PDE7B overexpression plasmid in Hep3B2.1-7 cells. **(C)** Transwell assays were employed to evaluate the migration and invasion capabilities of PDE7B-overexpressing plasmid-transfected Hep3B2.1-7 cells. Scale bar, 200 μm. **(D)** Wound-healing assays assessed the migration of Hep3B2.1-7 cells transfected with PDE7B plasmids. Scale bar, 50 μm. **(E, F)** Colony formation and EdU proliferation assays were applied to assess the proliferation of Hep3B2.1-7 cells. Scale bar, 200 μm. **(G)** qRT−PCR was used to determine the efficiency of PDE7B siRNA in PLC/PRF/5 cells. **(H)** Transwell assays were used to detect the migration and invasion of PDE7B-knockdown PLC/PRF/5 cells. Scale bar, 200 μm. **(I)**. The influence of PDE7B knockdown on the migration of PLC/PRF/5 cells was evaluated via a wound healing assay. Scale bar, 50 μm. **(J, K)** Cell proliferation was assessed via colony formation and EdU proliferation assays following PDE7B knockdown in PLC/PRF/5 cells. Scale bar, 200 μm. Each experiment was conducted in triplicate. Statistical significance was determined using the Student’s t-test (*p<0.05).

We further explored the potential role of PDE7B in HCC through *in vitro* experiments. Compared to that in normal liver cells, the expression of PDE7B in HCC cell lines (PLC/PRF/5, LM3, Huh-7, Li-7, and Hep3B2.1-7) was significantly lower (**Figure 3A**). To assess the transfection efficiency, we transfected Hep3B2.1-7 cells with a plasmid that overexpresses PDE7B. Subsequently, we confirmed this by conducting qRT−PCR analysis to validate the transfection efficiency (**Figure 3B**). To evaluate the migratory and invasive capacities of HCC cells, experiments were performed utilizing the Transwell and the wound-healing assays. To assess the proliferation of HCC cells, we performed colony formation and EdU assays. By comparing the migration and invasion potentials of Hep3B2.1-7 cells overexpressing PDE7B to those of the control vector group, we observed a significant suppression of these two cell lines, as validated by Transwell and wound healing assays (**Figures 3C, D**). A colony formation assay demonstrated that the proliferative capacity of Hep3B2.1-7 cells was markedly decreased after PDE7B was overexpressed (**Figure 3E**). EdU assays demonstrated a decrease in the proliferative ability of PDE7B-overexpressing Hep3B2.1-7 cells compared to the control group (**Figure 3F**). The efficiency of PLC/PRF/5 cells transfected with siRNAs was assessed, which revealed a clear downregulation of PDE7B expression. (**Figure 3G**). The Transwell and wound healing experiments indicated a significant enhancement in the migration and invasion abilities of the PDE7B knockdown group compared to the control group (**Figures 3H, I**). Furthermore, inhibiting the expression of PDE7B in PLC/PRF/5 cells resulted in a decrease in the quantity of replicated PLC/PRF/5 cells as observed in the colony formation assay (**Figure 3J**). Moreover, the percentage of proliferative cells, as determined by the EdU assay, was notably higher in the PDE7B knockdown group compared to the control group (**Figure 3K**). In brief, PDE7B inhibited the migration, proliferation and invasion of HCC cells, and suppressed in the progression of hepatocarcinoma.

### 3.4 PDE7B induced cell apoptosis via reactive oxygen species levels and affected signaling in HCC cells

**Figure 4:**
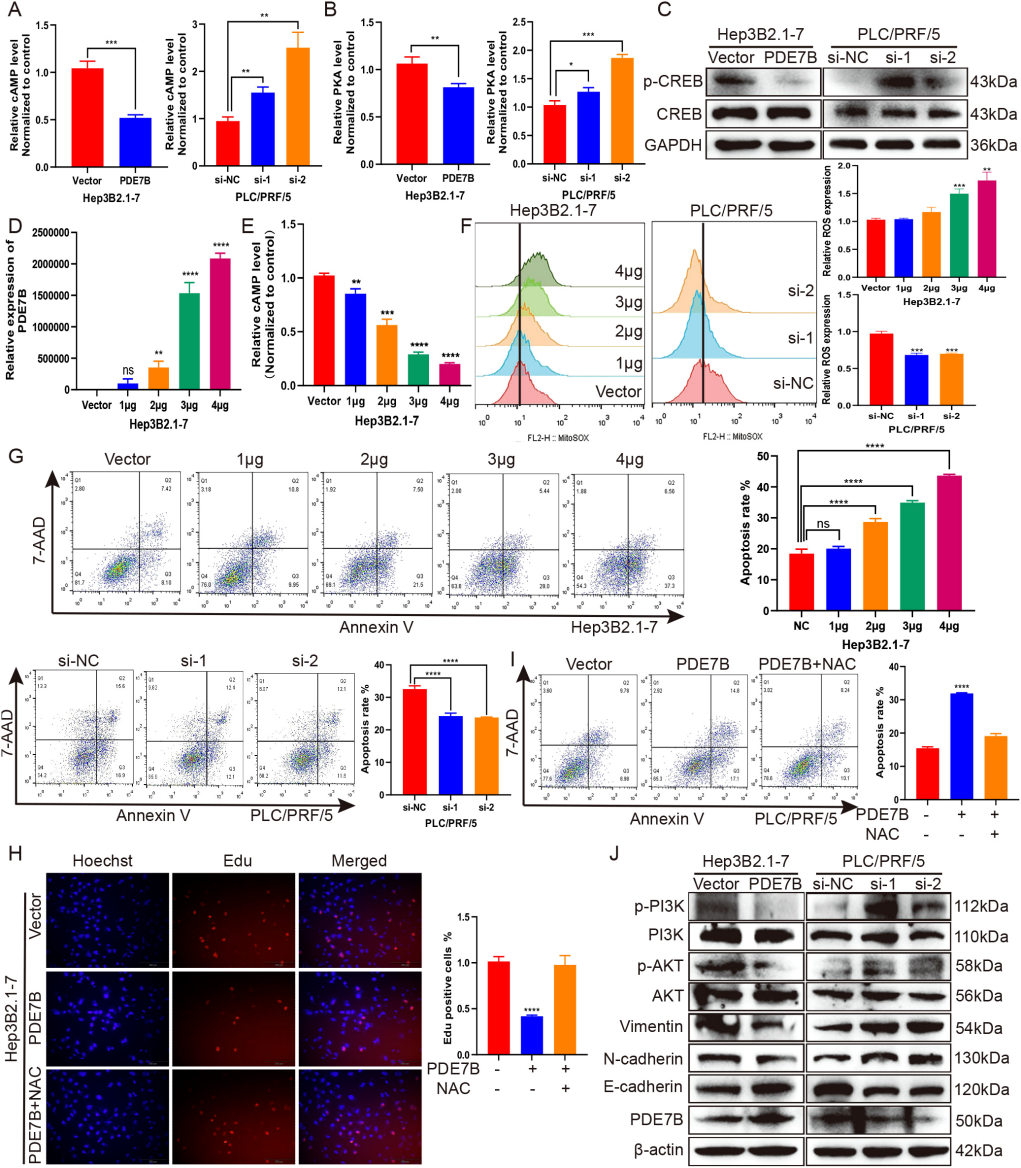
PDE7B affects cell apoptosis via reactive oxygen species (ROS) levels and signaling in HCC cells. **(A)** The level of intracellular cAMP was detected via a cyclic AMP assay kit after overexpressing PDE7B in Hep3B2.1-7 cells or knocking down PDE7B in PLC/PRF/5 cells. **(B)** A colorimetric activity kit was used to assess the activity of PKA after overexpressing PDE7B in Hep3B2.1-7 cells or knocking down PDE7B in PLC/PRF/5 cells. **(C)** The protein expression levels of CREB and p-CREB were measured via western blotting. **(D)** The level of PDE7B expression was determined via qRT−PCR after treatment with different doses of the overexpression plasmids. **(E)** Determination of intracellular cAMP expression levels according to different overexpression plasmid doses. **(F)** A MitoSOX assay was performed to evaluate the mitochondrial ROS level. FlowJo software was used to calculate the average fluorescence intensity, with the control group being normalized to determine the values for other data sets. **(G)** Flow cytometry analysis was also conducted to assess the apoptosis rate of Hep3B2.1-7 and PLC/PRF/5 cells. **(H, I)** An EdU assay and flow cytometry were utilized to assess the proliferation and apoptosis of Hep3B2.1-7 cells after 48 hours of treatment with the vector or N-acetyl-L-cysteine. **(J)** The levels of PDE7B, N-cadherin, E-cadherin, Vimentin, p-AKT, AKT, p-PI3K, and PI3K were detected via western blotting in cells with PDE7B overexpression or knockdown. Student’s t-test was employed to determine statistical significance (*p<0.05).

To elucidate the molecular mechanism by which PDE7B impacts HCC cells, we stably overexpressed PDE7B in Hep3B2.1-7 cells and knocked down PDE7B in PLC/PRF/5 cells. Compared with that in the control vector group, PDE7B overexpression in Hep3B2.1-7 cells resulted in a significant decrease in cAMP levels. Knockdown of PDE7B resulted in elevated levels of cAMP compared to the control group (**Figure 4A**). PKA participates in the cAMP signaling pathway in cells and is believed to have a crucial role in the pathological process of tumor development (25). Therefore, we hypothesized that PDE7B may regulate cAMP dependent PKA activity by altering the intracellular cAMP level. To verify this, colorimetric activity kit was used to investigate the impact of PDE7B on PKA activity. PDE7B overexpression in Hep3B2.1-7 cells significantly suppressed PKA activity compared to the control group. PDE7B knockdown elevated PKA activity in PLC/PRF/5 cells (**Figure 4B**). The expression of multiple oncogenes is regulated via an essential transcription factor, cAMP response element-binding protein (CREB) which acts as a target protein of PKA (26). The phosphorylation of CREB was consequently decreased when PDE7B was overexpressed in Hep3B2.1-7 cells and increased when PDE7B was knocked down in PLC/PRF/5 cells via western blotting (**Figure 4C**). So, by regulating the cAMP/PKA/CREB pathway, PDE7B affects the malignant growth of HCC cells.

The cAMP/PKA/CREB signaling pathway may induce cell apoptosis by regulating mitochondrial ROS levels, thereby affecting the progression of HCC (27). The impact of different doses of the overexpression agent were validated via qRT−PCR (**Figure 4D**). The expression level of intracellular cAMP decreased as the concentration of the overexpression plasmid increased (**Figure 4E**). To confirm this hypothesis, a MitoSOX assay was used to determine that the mitochondrial ROS concentration increased with increasing PDE7B overexpression in Hep3B2.1-7 cells, while the ROS concentration decreased when PDE7B was knocked down in the PLC/PRF/5 cell (**Figure 4F**). Along with the increase in PDE7B expression in Hep3B2.1-7 cells, cell apoptosis was subsequently enhanced, as determined via flow cytometry analysis. In contrast to PDE7B overexpression, PDE7B knockdown in PLC/PRF/5 cells decreased apoptosis (**Figure 4G**). These outcomes indicated that oxidative stress mediated the inhibitory impact of PDE7B on the growth and survival of HCC cells. N-acetylcysteine (NAC) was used to eliminate ROS to confirm this hypothesis. After NAC treatment, the decline in cell viability induced by PDE7B overexpression was counteracted by promoting cell proliferation and inhibiting apoptosis in Hep3B2.1-7 cells (**Figures 4H, I**). These findings suggest that PDE7B’s suppressive effect on HCC cell viability stems from its ability to inhibit oxidative stress.

Additionally, western blotting demonstrated that PDE7B protein expression was strongly promoted when PDE7B was overexpressed and decreased when PDE7B was knocked down. The EMT pathway is pivotal in tumor migration and invasion. PDE7B overexpression led to reduced N-cadherin and Vimentin expression while increasing E-cadherin protein levels. The opposite effect was observed when PDE7B was knocked down. Moreover, a large amount of literature indicates that ROS are inhibitors of the PI3K/AKT signaling pathway. We hypothesized that PDE7B may also regulate liver cancer progression via the PI3K/AKT signaling pathway. PDE7B overexpression in Hep3B2.1-7 cells inhibited the protein expression of phosphorylated AKT and phosphorylated PI3K. In contrast, phosphorylated AKT and phosphorylated PI3K were promoted when PDE7B was silenced in PLC/PRF/5 cells (**Figure 4J**).

### 3.5 PDE7B overexpression inhibited tumor growth in HCC *in vivo*

**Figure 5:**
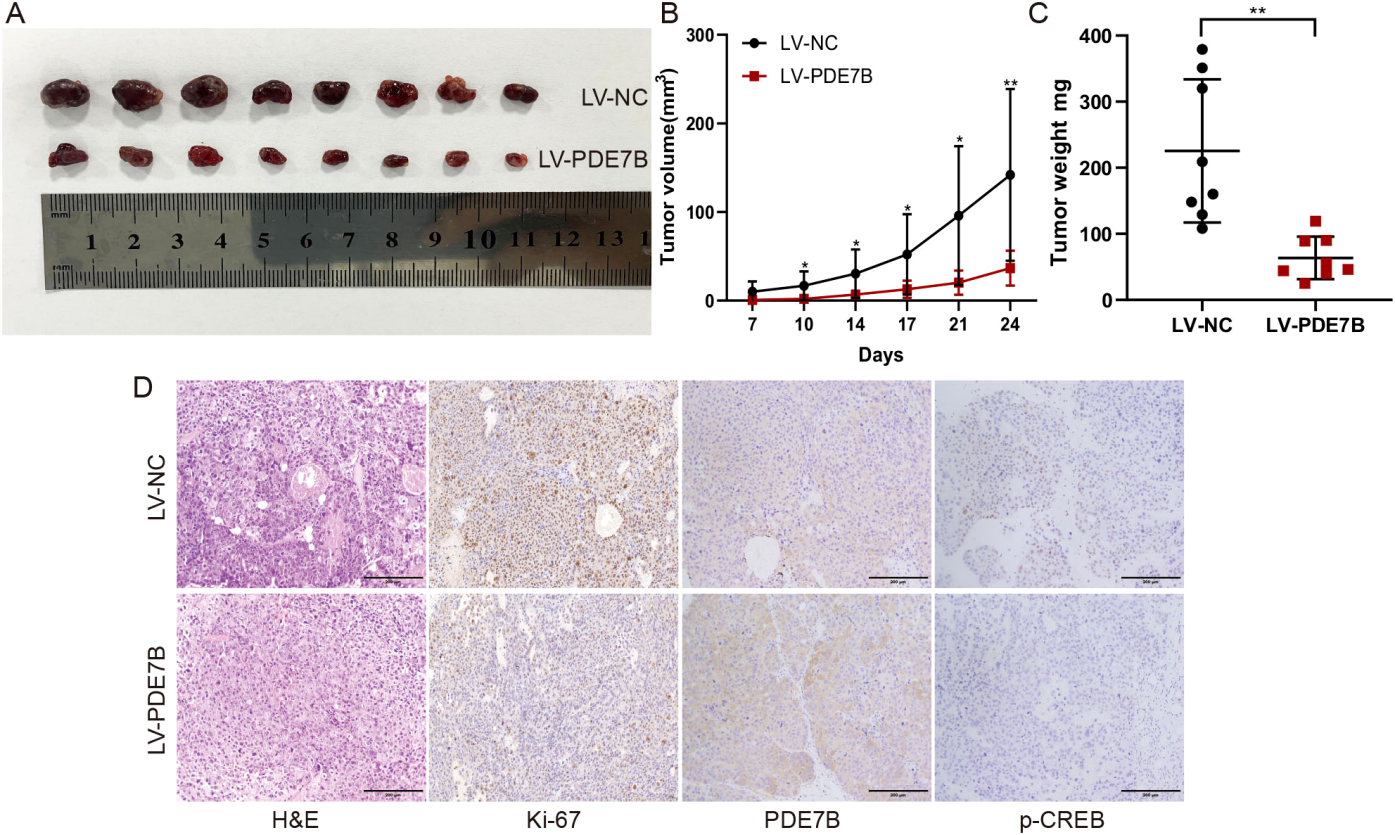
Overexpression of PDE7B inhibits tumor growth *in vivo*. **(A)** Effect of PDE7B on the growth of hepatocellular carcinoma tumors after hypodermic injection. Calculations were performed to determine the volume **(B)** and weight **(C)** of the tumors obtained from the mice. **(D)** H&E staining was used to reveal the influence of liver tissue PDE7B in the LV-NC and LV-PDE7B groups, while immunohistochemistry was conducted to assess Ki67 protein expression, PDE7B, and p-CREB. Two tailed Student’s t-test was employed to determine statistical significance (*p<0.05).

Cells overexpressing PDE7B were inoculated subcutaneously into nude mice to validate the tumorigenic impact of PDE7B *in vivo*. Tumor weight was assessed after 4 weeks by removing the subcutaneous tumor and measuring the tumor volume every 3-4 days in cells overexpressing PDE7B. Mice treated with LV-PDE7B exhibited notable reductions in both tumor volume and weight compared to the control group (**Figures 5A–C**). Furthermore, compared to the control group, the LV-PDE7B group displayed a decrease in tumor cell count, as evidenced by H&E staining analysis. Overexpression of PDE7B led to decreased protein levels of Ki67 and p-CREB, while increasing PDE7B protein levels, as determined by IHC analysis (**Figure 5D**). In brief, PDE7B overexpression inhibits tumor development *in vivo*.

### 3.6 The relationship between Sorafenib and PDE7B in inhibiting tumor growth

**Figure 6:**
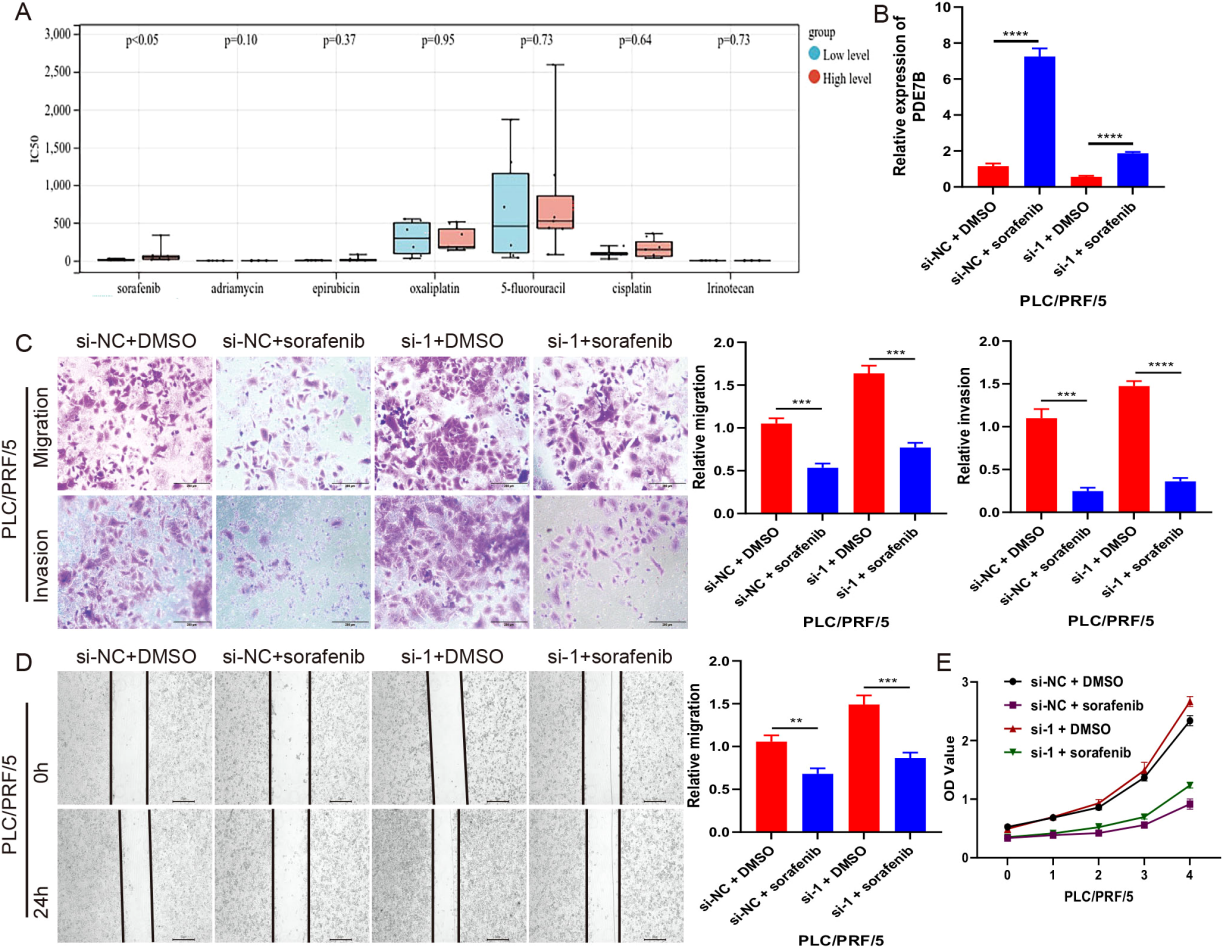
The relationship between Sorafenib and PDE7B in inhibiting tumor growth. **(A)** Drug sensitivity of 7 chemotherapy drugs in HCC in the database. **(B)** PDE7B expression levels were assessed via qRT-PCR in sorafenib-treated PLC/PRF/5 cells. transwell **(C)**, scratch **(D)**, and CCK-8 **(E)** assays were employed to measure PLC/PRF/5 growth cells after sorafenib treatment. Statistical significance was determined using the two tailed Student’s t-test (*p<0.05).

To refine clinical treatment strategies, we screened seven major chemotherapeutic drugs from the database (**Figure 6A**), among which sorafenib was the first targeted drug approved for the treatment of advanced HCC and was currently one of the primary drugs used in clinical HCC treatment. PCR analysis revealed elevated levels of PDE7B in cells treated with sorafenib (**Figure 6B**). Cells treated with the Sorafenib solvent (DMSO) were used as the vehicle control group to ensure that the solvent itself did not affect the cells, eliminating interference from non-drug factors. Subsequently, Transwell (**Figure 6C**), scratch experiments (**Figure 6D**), and CCK-8 (**Figure 6E**) further confirmed that when PDE7B expression is reduced or inhibited, HCC cells tend to proliferate, invade, and metastasize more easily. However, sorafenib’s antitumor effect may be enhanced, potentially compensating for the tumor-promoting advantage brought about by low PDE7B expression.

### 3.7 The expression of PDCD1 is related to the expression of PDE7B in HCC

**Figure 7:**
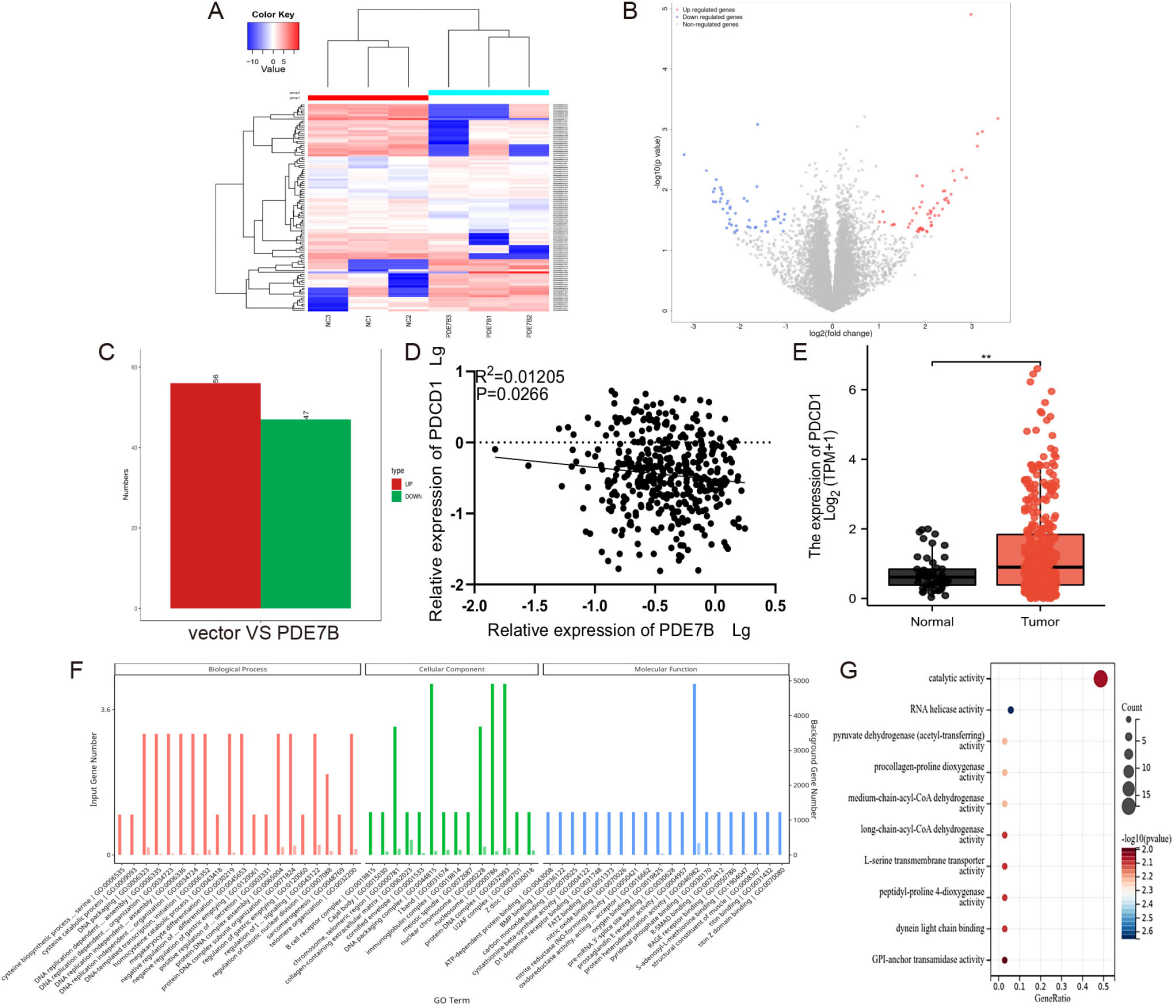
PDE7B affects the development of hepatocellular carcinoma through regulating PDCD1. **(A)** A heatmap illustrating the gene expression patterns in Hep3B2.1-7 cells subjected to vector plasmid transfection was generated, as was the case for PDE7B plasmid transfected cells. **(B)** The volcano plot illustrates differential expression of genes. **(C)** RNA sequencing of the differentially expressed upregulated and downregulated genes. **(D)** The expression levels of PDE7B and PDCD1 in HCC tissues were examined using qRT-PCR alongside a Pearson correlation analysis to assess their relationship. **(E)** The expression of PDCD1 in HCC on the basis of data from the TCGA database. **(F)** GO analysis of the PDE7B target genes revealed enrichment in the BP, CC, and MF categories. **(G)** KEGG bubble plot of the enrichment of the different PDE7B target genes (*p<0.05).

To explore the downstream effects of PDE7B and unravel its molecular mechanism, we performed an RNA sequencing analysis to investigate the potential target genes involved. A comparison between the vector group and the PDE7B group was performed to evaluate the differences in gene expression. This evaluation involved the use of heatmaps (**Figure 7A**) and volcano plots (**Figure 7B**). According to the results obtained from the RNA-sequencing analysis, compared with those in the vector cohort, 56 genes in the PDE7B cohort exhibited upregulated expression, and 47 genes exhibited downregulated expression (**Figure 7C**). After conducting a thorough analysis of the transcriptome sequencing data, a notable finding emerged regarding the impact of overexpressing PDE7B on gene expression. Specifically, it was found that the gene PDCD1 exhibited the most significant up-regulation compared to other differentially expressed genes. This indicates the crucial role of PDE7B in regulating the expression of PDCD1, indicating a potential mechanism by which PDE7B influences cellular processes. We found that PDCD1 expression was negatively correlated with PDE7B expression in HCC tissues (**Figure 7D**). According to the TCGA database, there was significant upregulation of PDCD1 in HCC tissue compared to adjacent normal tissue samples (**Figure 7E**). Further investigation into the specific interactions between PDE7B and PDCD1 may provide valuable insights into their functional relationship and implications for therapeutic interventions targeting these genes. These findings suggest PDCD1 as a potential target of PDE7B in HCC. GO analysis of the target genes indicated that PDE7B could participate in DNA replication, regulate transcription, and engage in chemical modifications (**Figure 7F**). A thorough analysis of the KEGG signaling pathway demonstrated marked enrichment of genes related to apoptosis, the cAMP pathway, and oxidative stress (**Figure 7G**). These findings suggest that PDE7B may inhibit HCC development by affecting PDCD1.

## 4 Discussion

The PDE7 family is currently in the initial stages of comprehension, and although PDE7B has been recognized as a component of the PDE7 family, its specific function has not been determined (28). Previous research has shown that PDE7B contributes to the development of multiple disorders. Zhang et al. indicated that inhibiting PDE7B can lead to increased cAMP levels, which in turn promotes apoptosis in chronic lymphocytic leukaemia cells (29). The study by Brooks MD et al. confirmed that the expression of PDE7B is regulated by human brain microvascular endothelial cells (HBMECs). This regulation is primarily achieved through direct cell-cell contact rather than relying on secreted factors. PDE7B plays a crucial role in the tumor microenvironment, and the presence of endothelial cells may promote the expression of PDE7B, thereby influencing the occurrence and development of glioblastoma (30). Zhang et al. confirmed that PDE7B regulates the proliferation and migration of breast cancer cells by affecting intracellular cAMP levels through its interaction with miR-200c. Additionally, the inhibition of PDE7B enhances the sensitivity of breast cancer cells to certain chemotherapeutic agents, such as microtubule-targeting drugs, thereby improving the effectiveness of chemotherapy (31). In addition, PDE7B can also play a role in asthma, schizophrenia, Alzheimer’s disease and other diseases (32, 33).

However, few researches have reported the connection between PDE7B and cancer migration, invasion, and proliferation. As of now, no study has investigated the expression of PDE7B in HCC or its prognostic significance. So, we explored the expression and regulation of PDE7B in HCC. Moreover, we also wanted to determine the potential of PDE7B in combating HCC tumors. Hence, we analyzed the antitumor effects of PDE7B in liver cancer. In this study, PDE7B was found to be significantly downregulated in HCC by using an online database. In our investigation, compared with adjacent paracancerous tissue, tumor tissue from HCC patients exhibited a noteworthy decrease in PDE7B mRNA and protein levels. Moreover, we discovered that liver cancer clinical indicators are correlated with PDE7B expression and that HCC patients with elevated PDE7B expression have an improved prognosis. Subsequently, an investigation into the biological role of PDE7B in HCC was undertaken. By conducting experiments in controlled laboratory settings and in live organisms, the upregulation of PDE7B was shown to effectively hinder cell proliferation, invasion, and migration. These findings elucidated the tumor-suppressive properties of PDE7B in HCC.

The occurrence and development of tumors are significantly influenced by cAMP, as supported by a substantial body of research findings. As a second messenger in cell signal transduction, cAMP is mainly regulated by the PDE family. Studies have revealed that cancer cells generally exhibit low intracellular cAMP concentrations and PKA activity, indicating the crucial involvement of PDEs in cancer progression (34, 35). Therefore, we speculate that PDE7B may affect tumor progression by regulating cAMP levels. As a major target of cAMP, PKA regulates gene expression by phosphorylating substrates, such as CREB, thereby affecting processes like cell differentiation, proliferation, and apoptosis (36, 37). In our study, knocking out PDE7B reduced the hydrolysis of cAMP and activated PKA, which phosphorylated downstream PKA substrates such as CREB, leading to the invasion and migration of tumor cells.

In PDE7B-mediated oxidative stress, PDE7B’s activity likely affects mitochondrial function and the oxidative stress response by lowering cAMP levels, which promotes apoptosis. In contrast, the NRF2-KEAP1 axis primarily regulates the expression of antioxidant genes, protecting cells from oxidative stress by activating antioxidant defense mechanisms. The NRF2-KEAP1 axis is a well-established regulatory pathway for maintaining redox homeostasis. Under normal conditions, KEAP1 binds to NRF2, promoting its degradation. In response to oxidative or electrophilic stress, NRF2 dissociates from KEAP1 and translocates to the nucleus, where it activates the transcription of a range of antioxidant genes, such as glutathione peroxidase and superoxide dismutase. This axis is central to the cellular antioxidant defense system, countering oxidative damage by eliminating ROS and restoring cellular homeostasis (38, 39).

In this study, we hypothesize that PDE7B impacts tumor progression through the cAMP/PKA/CREB regulatory axis. The existing literature suggests that this regulatory axis may influence cell survival by affecting mitochondrial ROS levels in HCC cells (40–42). Additionally, multiple investigations have proven that the buildup of ROS impedes the PI3K/AKT signaling pathway. Our results demonstrated that overexpression of PDE7B leads to a significant increase in mitochondrial ROS, resulting in elevated cell apoptosis and suppressed proliferation of cancer cells. The effect of PDE7B can be eliminated by reducing ROS levels via the use of NAC, indicating that increased ROS levels serve as a key marker for the PDE7B-mediated inhibition of liver cancer cell apoptosis. Furthermore, our study demonstrated that upregulation of PDE7B inhibits cellular EMT and the PI3K/AKT signaling pathway. *In vivo* experiments confirmed that overexpression of PDE7B inhibits tumor growth. Finally, we found that sorafenib can upregulate the expression of PDE7B. Moreover, in the frontal knockout PLC/PRF/5 cell line treated with sorafenib, tumor invasion, migration, and proliferation were significantly attenuated.

Through transcriptome sequencing analysis, we identified PDCD1 as a potential target of PDE7B in hepatocellular carcinoma. Our findings suggest that PDE7B may impede the invasion and migration of HCC by suppressing PDCD1 expression. PDCD1, an immunoglobulin and the gene encoding PD-1, is typically present on activated B lymphocytes, CD4^+^/CD8^+^ T cells, myeloid cells, and natural killer cells (43–45). A recent study has also shown that tumor cells can express PDCD1, potentially aiding in immune evasion and enhancing invasive capabilities. This novel insight is crucial for unraveling the mechanisms behind tumor immune evasion (46, 47). Prior research has highlighted the significant involvement of miR-15/16 in chronic lymphocytic leukemia by targeting PDCD1 expression. The interaction between PD-1 and PD-L1 is a crucial mechanism employed by malignant cells to evade T cell immunity (48). In patients with stage II to stage III colon cancer, increased PDCD1 expression in cancer tissues hinders T cell immune responses to tumors (49). Investigation into PD-1 signaling and its role in promoting tumor-infiltrating myeloid-derived suppressor cells and gastric tumorigenesis in mice have shown that PD-1 expression on tumor or immune cells augments carcinogen-induced gastric tumors (50). Moreover, abnormal expression of PDCD1 has been observed in several cancers, including esophageal cancer (51), breast cancer (52), lung cancer (53), and thyroid cancer (54). The specific mechanism of PDCD1’s action on tumors remains unclear and requires further investigation. Our current findings suggest that PDCD1 is upregulated in hepatocellular carcinoma and shows an inverse correlation with PDE7B expression. Increased PDE7B expression leads to the inhibition of PDCD1 expression, triggering immune suppression and restraining the invasion and migration abilities of tumor cells. These findings underscore the potential influence of PDE7B on the pathogenesis and advancement of hepatocellular carcinoma by modulating PDCD1.

While our study provides valuable insights, there are certain limitations that should be considered. This study used a single mouse model to investigate tumor growth *in vivo*, which is a limitation of our research. Although this model effectively simulates certain aspects of tumor growth, it does not fully represent the heterogeneity of tumors. Future studies should consider using multiple mouse models to explore tumor biology more comprehensively and to validate the conclusions drawn from this study, thereby improving the generalizability of the results.

**Figure 8:**
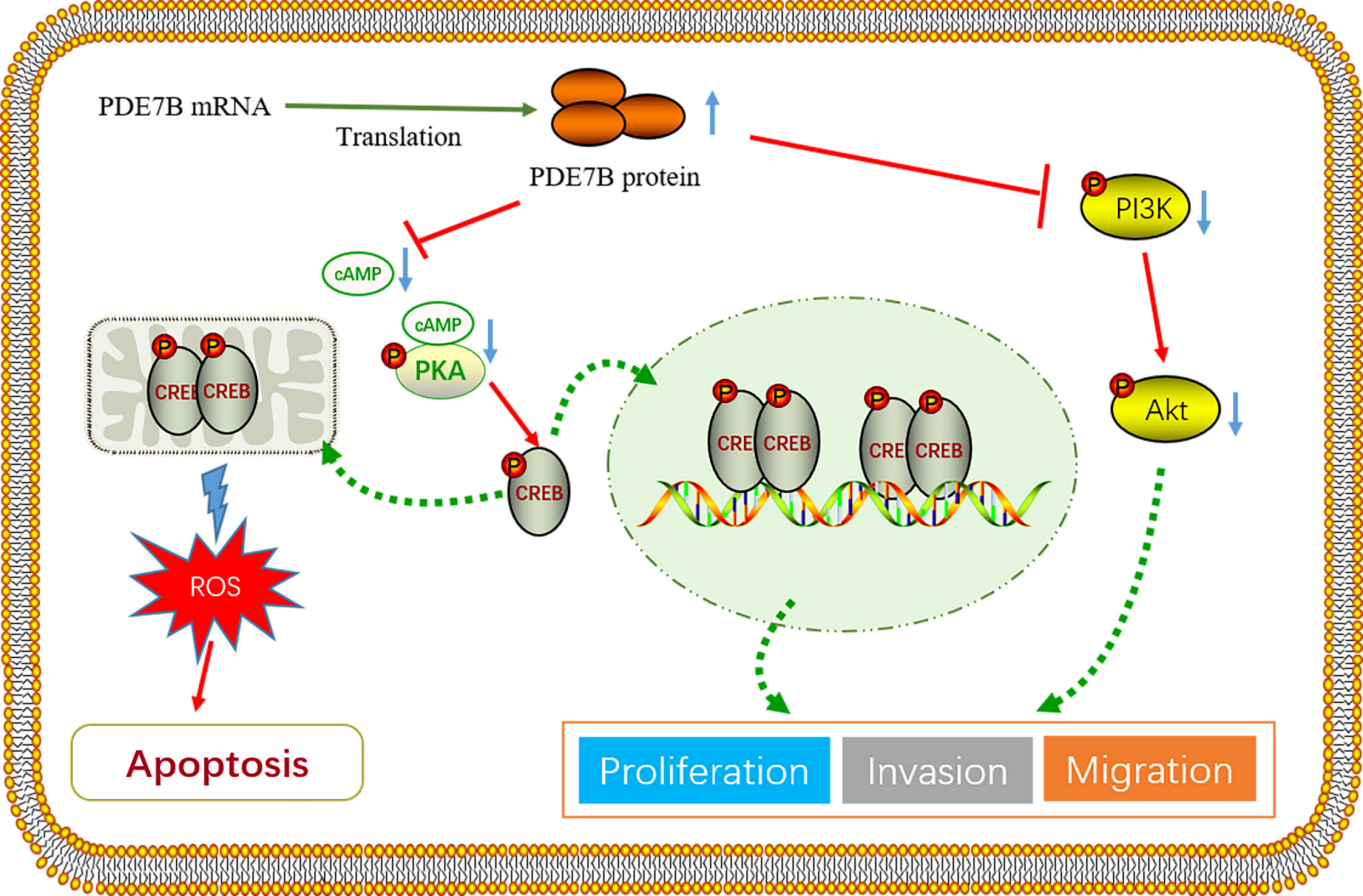
The regulatory mechanism of PDE7B in inhibiting HCC proliferation and metastasis.

Taken together, we have developed a hypothetical framework to consolidate our discoveries, illustrated in **Figure 8**. Our study demonstrated that PDE7B, which functions as a tumor suppressor gene, significantly contributes to the initiation and progression of hepatocellular carcinoma. Patients exhibiting low levels of PDE7B expression tend to have a poorer prognosis. Conversely, high expression of PDE7B appears to inhibit the progression of HCC. The crucial role of the PDE7B/PKA/cAMP axis in repressing the malignant traits of HCC cells emphasizes the significance of PDE7B as a potential therapeutic target. Furthermore, PDE7B has the potential to be effectively integrated with current targeted therapies and immunotherapies to improve overall treatment effectiveness. Its function as a possible biomarker for diagnosing, predicting prognosis, and assessing treatment responses in HCC emphasizes its significance in clinical practice. Nevertheless, the use of PDE7B in medical applications requires additional investigation. In the future, we will explore the targeting of PDE7B in cancer therapy and conduct further preclinical and clinical studies to validate its role in tumor progression.

## 5 Conclusion

This investigation highlights the correlation between decreased PDE7B expression in hepatocellular carcinoma and unfavorable prognosis. Furthermore, PDE7B might collaboratively enhance the impact of sorafenib in combination with chemotherapy via a specific mechanism, and thus could serve as the promising biomarker and the therapeutic target for the prognosis and immunotherapy of HCC. The molecular underpinnings of PDE7B in HCC necessitate additional experimentation for comprehensive elucidation.

## Data Availability

The datasets presented in this study can be found in online repositories. The names of the repository/repositories and accession number(s) can be found below: https://www.ncbi.nlm.nih.gov/, PRJNA1131143.
