# Supplementary material for: The mechanism of PDE7B inhibiting the development of hepatocellular carcinoma through oxidative stress
Source: Front Immunol. 2024 Nov 21;15:1469740. doi: 10.3389/fimmu.2024.1469740 (PMC11617559; doi:10.3389/fimmu.2024.1469740)
Supplement: Supplementary file 1 [file DataSheet1.pdf]

Supplementary Material

1 Supplementary Figures

The entire original blots are submitted as part of the supplementary material.

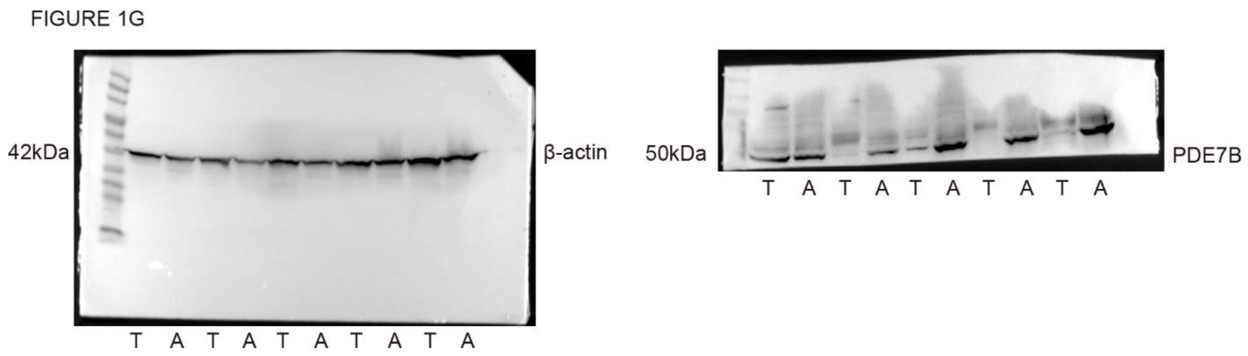

Supplementary Figure 1. The entire original blots of FIGURE 1G.

FIGURE 4C

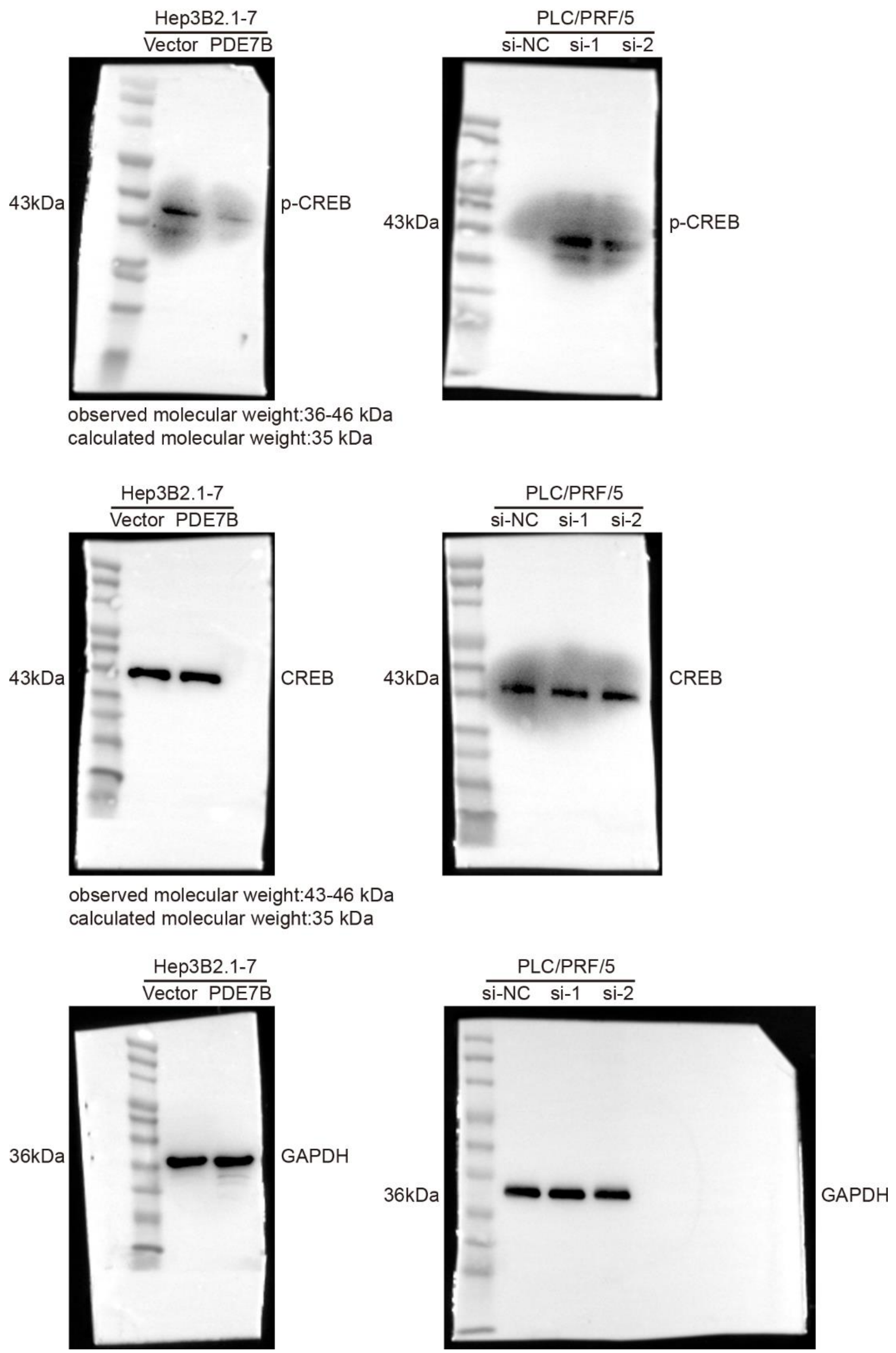

Supplementary Figure 2. The entire original blots of FIGURE 4C.

FIGURE 4J

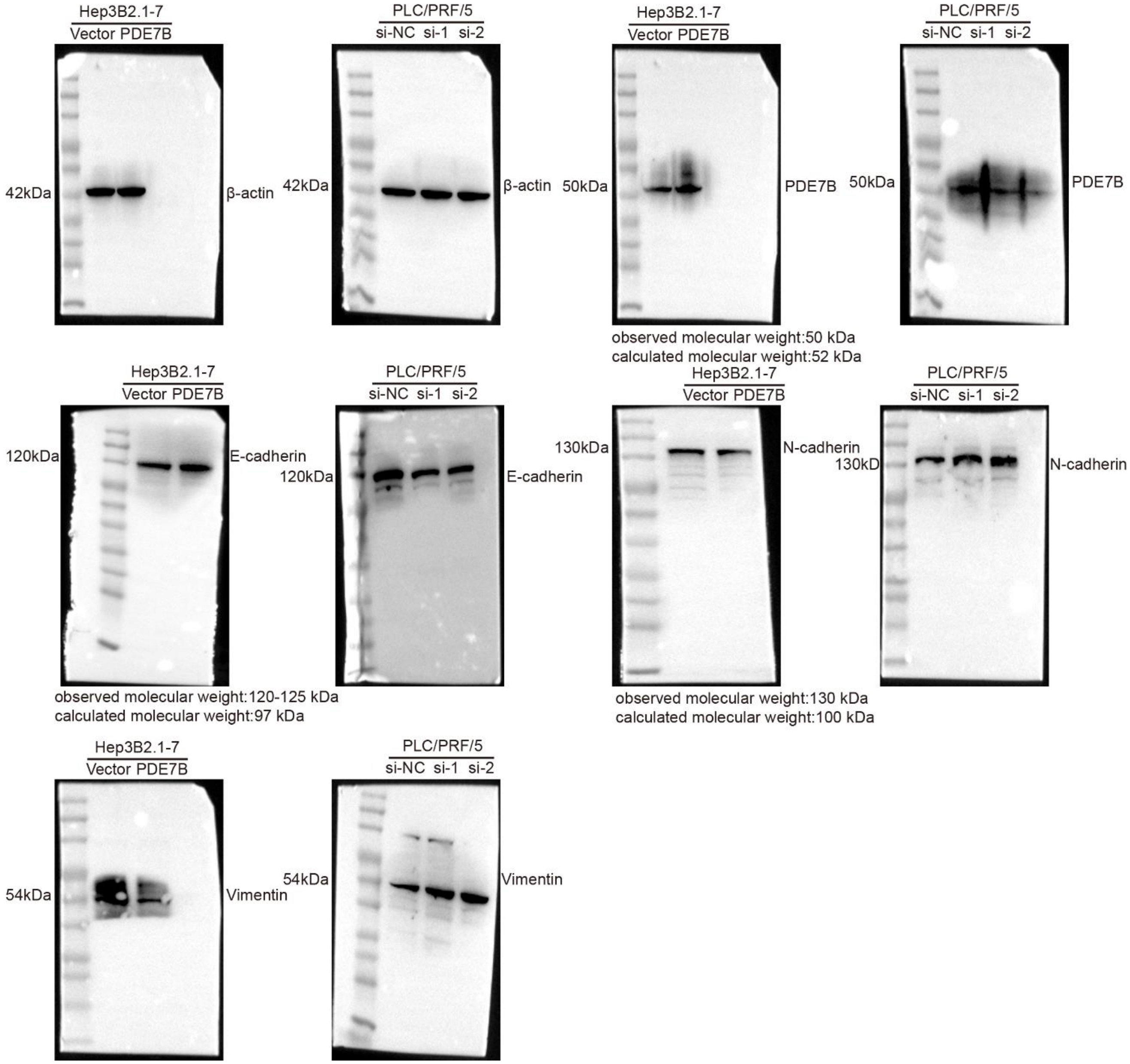

Supplementary Figure 3. The entire original blots of FIGURE 4J-1.

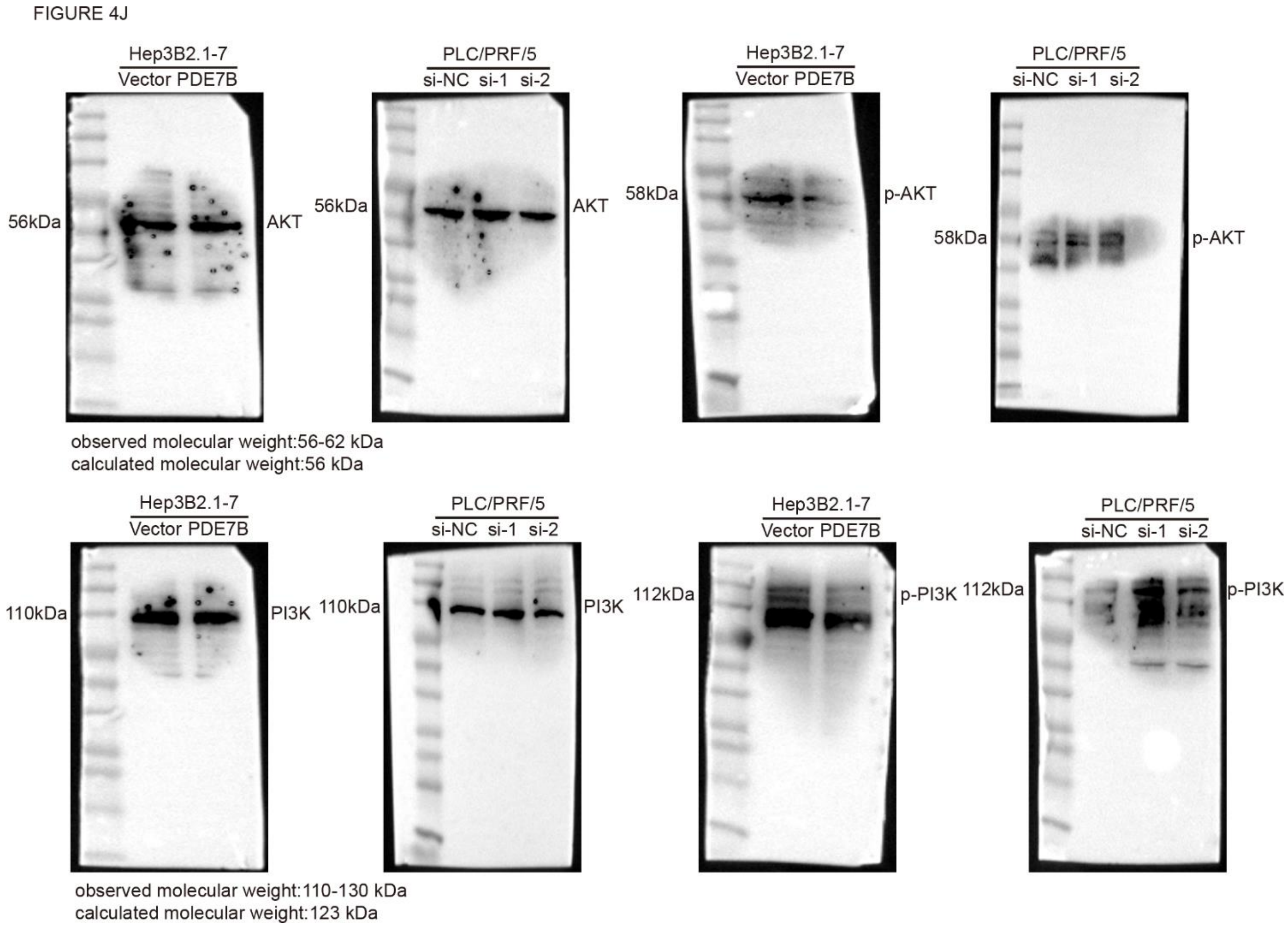

Supplementary Figure 4. The entire original blots of FIGURE 4J-2.

A

	sense(5’-3’)	antisense(5’-3’)
PDE7B-Homo-547（siRNA-1）	GCUGCUUCGUGGAAUUAUATT	UAUAAUUCCACGAAGCAGCTT
PDE7B-Homo-1466（siRNA-2）	GCCCAUUUCACGGGUAACATT	UGUUACCCGUGAAAUGGGCTT

B

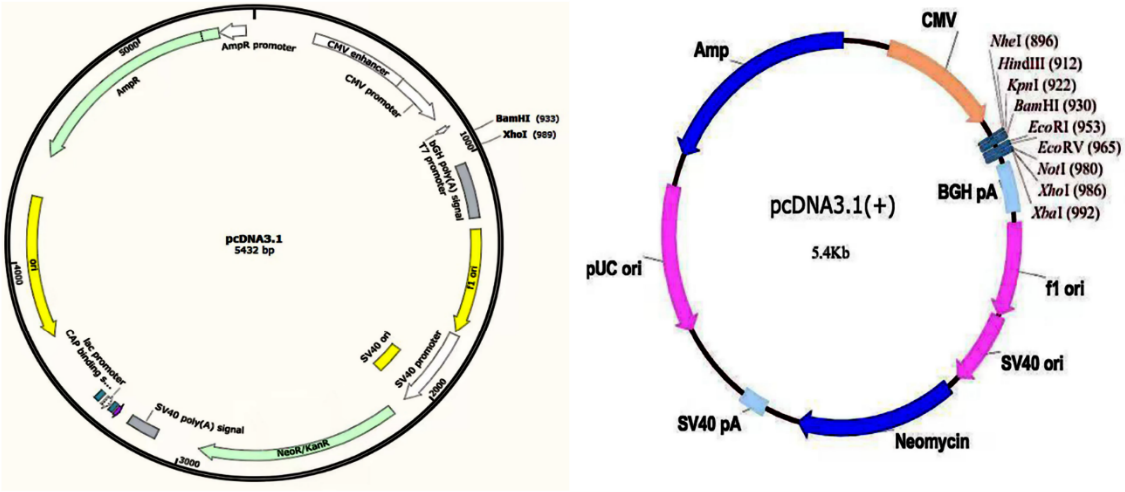

C

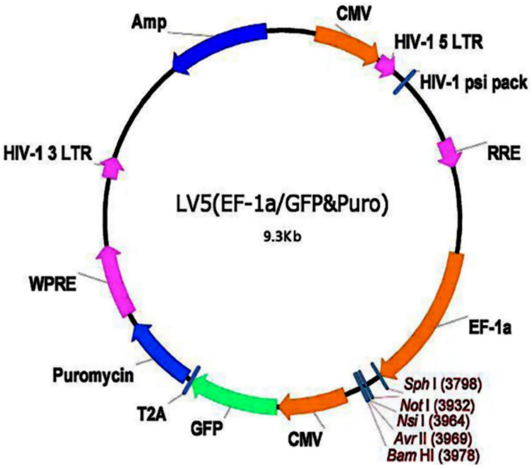

Supplementary Figure 5. (A) The gene fragments of the siRNAs. (B) The map of PDE7B overexpression plasmid. (C) The map of PDE7B lentiviral vector.
